# The effect of vitamin B supplementation on neuronal injury in people living with HIV: a randomized controlled trial

**DOI:** 10.1093/braincomms/fcac259

**Published:** 2022-10-15

**Authors:** Erika Tyrberg, Lars Hagberg, Lars-Magnus Andersson, Staffan Nilsson, Aylin Yilmaz, Åsa Mellgren, Kaj Blennow, Henrik Zetterberg, Magnus Gisslén

**Affiliations:** Department of Infectious Diseases, Institute of Biomedicine, The Sahlgrenska Academy at the University of Gothenburg, Gothenburg, Sweden; Department of Infectious Diseases, Sahlgrenska University Hospital, Gothenburg, Sweden; Department of Infectious Diseases, Institute of Biomedicine, The Sahlgrenska Academy at the University of Gothenburg, Gothenburg, Sweden; Department of Infectious Diseases, Sahlgrenska University Hospital, Gothenburg, Sweden; Department of Infectious Diseases, Institute of Biomedicine, The Sahlgrenska Academy at the University of Gothenburg, Gothenburg, Sweden; Department of Infectious Diseases, Sahlgrenska University Hospital, Gothenburg, Sweden; Department of Mathematical Sciences, Chalmers University of Technology, Gothenburg, Sweden; Department of Laboratory Medicine, Institute of Biomedicine, University of Gothenburg, Gothenburg, Sweden; Department of Infectious Diseases, Institute of Biomedicine, The Sahlgrenska Academy at the University of Gothenburg, Gothenburg, Sweden; Department of Infectious Diseases, Sahlgrenska University Hospital, Gothenburg, Sweden; Department of Infectious Diseases, Institute of Biomedicine, The Sahlgrenska Academy at the University of Gothenburg, Gothenburg, Sweden; Department of Infectious Diseases, Sahlgrenska University Hospital, Gothenburg, Sweden; Department of Psychiatry and Neurochemistry, Institute of Neuroscience and Physiology, The Sahlgrenska Academy at the University of Gothenburg, Mölndal, Sweden; Clinical Neurochemistry Laboratory, Sahlgrenska University Hospital, Mölndal, Sweden; Department of Psychiatry and Neurochemistry, Institute of Neuroscience and Physiology, The Sahlgrenska Academy at the University of Gothenburg, Mölndal, Sweden; Clinical Neurochemistry Laboratory, Sahlgrenska University Hospital, Mölndal, Sweden; Department of Neurodegenerative Disease, UCL Institute of Neurology, Queen Square, London, UK; UK Dementia Research Institute at UCL, London, UK; Hong Kong Center for Neurodegenerative Diseases, Hong Kong, China; Department of Infectious Diseases, Institute of Biomedicine, The Sahlgrenska Academy at the University of Gothenburg, Gothenburg, Sweden; Department of Infectious Diseases, Sahlgrenska University Hospital, Gothenburg, Sweden

**Keywords:** HIV, homocysteine, neurofilament light protein, B vitamins

## Abstract

Effective antiretroviral therapy has radically changed the course of the HIV pandemic. However, despite efficient therapy, milder forms of neurocognitive symptoms are still present in people living with HIV. Plasma homocysteine is a marker of vitamin B deficiency and has been associated with cognitive impairment. People living with HIV have higher homocysteine concentrations than HIV-negative controls, and we have previously found an association between plasma homocysteine concentration and CSF concentration of neurofilament light protein, a sensitive marker for ongoing neuronal injury in HIV. This prompted us to perform this randomized controlled trial, to evaluate the effect of vitamin B supplementation on neuronal injury in a cohort of people living with HIV on stable antiretroviral therapy. At the Department of Infectious Diseases at Sahlgrenska University Hospital in Gothenburg, Sweden, 124 virally suppressed people living with HIV were screened to determine eligibility for this study. Sixty-one fulfilled the inclusion criteria by having plasma homocysteine levels at or above 12 μmol/l. They were randomized (1:1) to either active treatment (with cyanocobalamin 0.5 mg, folic acid 0.8 mg and pyridoxine 3.0 mg) q.d. or to a control arm with a cross over to active treatment after 12 months. Cognitive function was measured repeatedly during the trial, which ran for 24 months. We found a significant correlation between plasma neurofilament light protein and plasma homocysteine at screening (*n* = 124, *r* = 0.35, *P* < 0.0001). Plasma homocysteine levels decreased by 35% from a geometric mean of 15.7 μmol/l (95% confidence interval 14.7–16.7) to 10.3 μmol/l (95% confidence interval 9.3–11.3) in the active treatment arm between baseline and Month 12. No significant change was detected in the control arm during the same time period [geometric mean 15.2 (95% confidence interval 14.3–16.2) versus geometric mean 16.5 μmol/l (95% confidence interval 14.7–18.6)]. A significant difference in change in plasma homocysteine levels was seen between arms at 12 months [−40% (95% confidence interval −48 to −30%), *P* < 0.001]. However, no difference between arms was seen in either plasma neurofilament light protein levels [−6.5% (−20 to 9%), *P* = 0.39], or cognitive measures [−0.08 (−0.33 to 0.17), *P* = 0.53]. Our results do not support a vitamin B–dependent cause of the correlation between neurofilament light protein and homocysteine. Additional studies are needed to further elucidate this matter.

## Teaser

Tyrberg *et al*. report the results of a randomized controlled trial investigating the effect of vitamin B supplementation on neuronal injury in people living with HIV with effective antiretroviral therapy. Supplementation decreased levels of homocysteine but not neuronal injury measured by neurofilament light protein.

## Graphical Abstract

**Graphical abstract:**
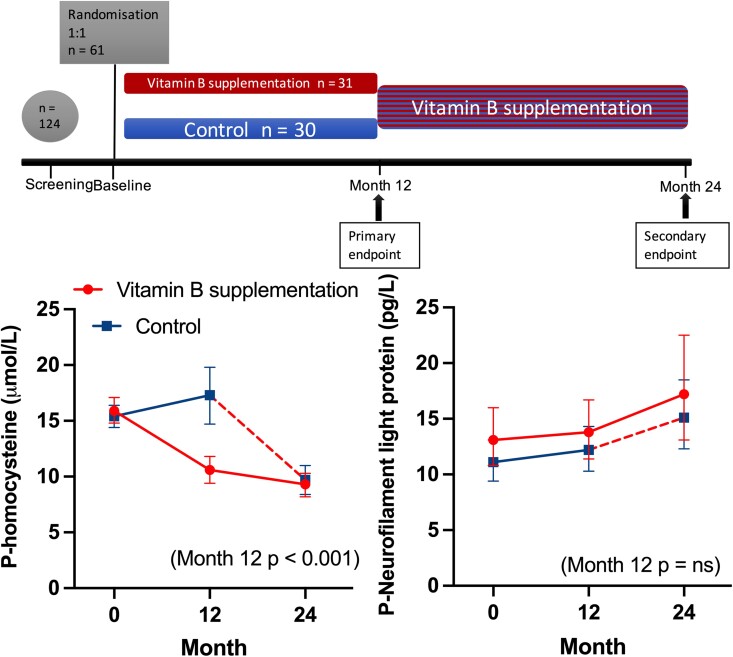

## Introduction

Before the introduction of effective antiretroviral therapy (ART), a significant number of people living with HIV (PLHIV) developed HIV-associated dementia (HAD), predominantly in the later stages of the disease.^1^ Since effective ART hinders the development of HAD, it has become rare, although milder forms of neurocognitive impairment are still present in PLHIV.^2^ These milder forms of HIV-associated neurocognitive disorders are divided into asymptomatic neurocognitive impairment (ANI) and HIV-associated mild neurocognitive disorders by the Frascati criteria.^3^ A few previous studies have indicated that PLHIV with ANI may have an increased risk of developing symptomatic cognitive impairment.^4,5^ However, numerous non-HIV-related confounders and contributors are present when using solely cognitive testing as a diagnostic tool. As a result, the clinical relevance of ANI is under debate.^6,7^

In addition, biochemical signs of ongoing neuronal injury are prevalent in untreated neuroasymptomatic PLHIV, as measured by neurofilament light protein (NfL).^8^ NfL levels are correlated to the progress of the HIV infection in untreated patients, and the highest levels of NfL are found in those with HAD.^8–11^ NfL, a subunit of the neurofilament protein in myelinated neurons, is to date the most sensitive biomarker of ongoing axonal injury in HIV.^11^ ART reduces levels of NfL,^12^ yet NfL levels remain higher after viral suppression by ART in PLHIV when compared with HIV-negative controls, and in some cases, they are above age-dependent cut-off levels.^9,13^ Data suggest that a rise in NfL precedes the onset of symptoms in untreated HIV, and thus may be used as a predictive marker.^14^ Initially, NfL could only be measured in CSF, but currently it is possible to analyse it in plasma. NfL concentrations are 50–100 times lower in plasma in comparison with CSF, and a relatively strong correlation can be found between plasma and CSF levels.^15–17^

Vitamin B_12_ deficiency may present with neurological symptoms, with or without haematological anomalies.^18,19^ In addition, folate deficiency may also give rise to neurological symptoms.^20,21^ Interestingly, HIV-associated myelopathy has a pathological resemblance to B_12_ deficiency.^22^ Vitamin B_12_ and folate are closely related to the metabolism of homocysteine. Homocysteine levels increase when vitamin B_12_ or folate levels are low, and therefore, homocysteine serves as a marker of vitamin B_12_ and folate deficiency.^23^ Elevated homocysteine levels are associated with cognitive impairment and the risk of developing Alzheimer’s disease.^24–26^ A number of studies have found this association to be independent of vitamin B_12_ and folate status.^27,28^ However, randomized placebo-controlled trials of vitamin B supplementation that effectively reduce homocysteine levels in plasma have been inconclusive regarding their effect on cognitive function in HIV-negative elderly individuals with or without cognitive impairment.^29–32^

A meta-analysis found that PLHIV have higher P-homocysteine levels than HIV-negative controls, and that P-homocysteine was higher in those on ART compared with untreated PLHIV.^33^ This may render PLHIV more vulnerable to pathologies related to homocysteine than the general population, making this a serious concern even in the era of successful ART.

We found an independent association between P-homocysteine and NfL concentrations in CSF in PLHIV in a previous study after adjusting for age, CD4 count^+^and CSF neopterin level.^34^ This prompted us to perform the present randomized controlled trial to evaluate the effect of vitamin B supplementation on neuronal injury in a cohort of PLHIV who were on stable ART.

## Materials and methods

### Study design

PLHIV on stable ART attending the out-patient clinic at the Department of Infectious Diseases, Sahlgrenska University Hospital, Gothenburg, Sweden, were eligible to participate in this single-centre, open, randomized, controlled trial with modified cross-over design. PLHIV who met the inclusion criteria (i.e. stable ART > 12 months, HIV-RNA < 50 copies/ml and age ≥18 years) and were not subject to any of the exclusion criteria were asked to participate at their regular follow-up visit. The exclusion criteria were treatment with trimethoprim-sulfamethoxazole or methotrexate, ongoing vitamin B_12_, B_6_, or folic acid supplementation, anti-epileptic treatment, small bowel or ventricular resection, absorption disturbance in the small bowel, ongoing neurological or severe psychiatric disease, history of a malignant tumour, severe ongoing or opportunistic infection, alcohol abuse, clinical depression, pregnancy, or significant vitamin B_12_ and/or folate deficiency that required higher treatment doses. Those who gave their consent were screened for participation in the trial.

**Figure 1:**
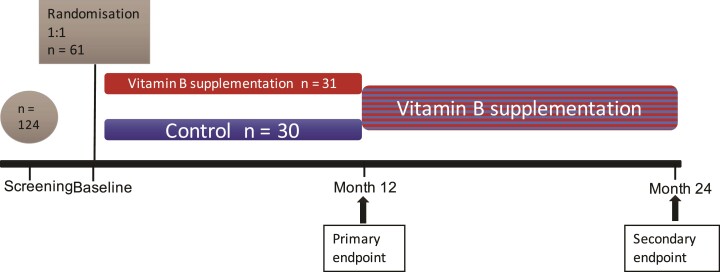
**Study design**.

Patients who had P-homocysteine levels ≥12 μmol/l were enrolled in the study at a baseline visit and randomized (1:1) to active treatment or control arm using a computer random number generator with a block size of 10. The allocation sequence was concealed in numbered envelopes (by E.T.). Enrolment was performed by several researchers (M.G., A.Y., L.M.A., L.H. and E.T.) all of whom were unaware of the allocation of previous participants. Those included in the active treatment arm received one tablet of TrioBe (Meda, Stockholm, Sweden), containing cyanocobalamin 0.5 mg, folic acid 0.8 mg and pyridoxine 3.0 mg q.d. All subjects were scheduled follow-up visits at months 1, 3, 6 and 12. Those in the control arm crossed over to the active treatment arm at the Month 12 visit. A modified cross-over design was used to enable both a longer follow-up time and a larger group that received the intervention. In addition, it facilitated recruitment and retention in study of the participants who were randomized to the control arm. All subjects were then followed until Month 24, with visits at Months 15 (control arm only), 18 and 24 (Fig. 1).

P-homocysteine, P-B_12_, P-folate, haemoglobin and creatinine levels were measured at each follow-up visit. P-NfL was analysed at initial screening and Months 3, 12 and 24. HIV-RNA levels and CD4^+^ counts were taken at baseline and Months 6, 12, 18 and 24. *S*-methylmalonic acid (S-MMA) was checked at baseline and Months 12 and 24. White blood cell count and mean corpuscular volume (MCV) were checked at baseline and Months 12 and 24. Signs of depression and alcohol use were checked at baseline and months 12 and 24 through the montgomery Åsberg depression rating scale (MADRS)^35^ and the alcohol use disorders identification test (AUDIT).^36^ Information on changes in concomitant medications, including food supplements, was obtained at every visit. In addition, adherence to ART and TrioBe treatment was checked at every visit as well as adverse effects. Study subjects were not allowed to take other medications or food supplements containing vitamin B_12_, B_6_, folic acid or medications that interact with these vitamins during the trial period.

Cognitive function was assessed by means of Cogstate (Cogstate Ltd, Melbourne, Australia) at baseline, and Months 12 and 24. Cogstate is a computerized neuropsychological test previously validated for HIV-associated neurocognitive impairment.^37,38^ Five tasks were performed testing five cognitive domains: detection (psychomotor function), identification (attention), one card learning (visual learning), one back test (working memory) and the Groton Maze learning test (executive function). The five test results were combined to give one total score (Cogstate combined *z*-score) that was used in the analysis.

The primary objective of the study was to determine the effect on P-NfL concentrations of treatment with B vitamins. The primary endpoint was set at 12 months of treatment and secondary endpoint at 24 months. The secondary objectives were (i) to determine the effect of vitamin B supplementation on neurocognitive performance, and (ii) to assess the relationship between P-NfL and P-homocysteine at screening.

### Laboratory assays

Plasma NfL concentration was measured using an in-house Single Molecule Array (Simoa) method on an HD-1 analyzer (Quanterix, Billerica, MA, USA), as previously described in detail.^15^

Clinical age-related cut-off levels for P-NfL were used: <18 years = <7 pg/ml; 18–50 years = <10 pg/ml; 51–60 years = <15 pg/ml; 61–70 years = <20 pg/ml; and > 70 years = <35 pg/ml.

Plasma homocysteine concentration was measured using the Roche Homocysteine Enzymatic Assay on a Cobas c501 instrument according to manufacturer’s instructions (Roche Diagnostics, Rotkreuz, Switzerland). P-B_12_ and P-folate was analysed on a Cobas e601 instrument, according to manufacturer’s instructions (Roche Diagnostics, Rotkreuz, Switzerland). The upper normal reference level (UNL) for P-homocysteine used by the laboratory was 15 μmol/l. No gold standard for the diagnosis of vitamin B_12_ or folate deficiency exists. Current reference values of the local laboratory are P-B_12_ 140–650 pmol/l, and 7–46 nmol/l for P-folate. The lower reference limits were used as a cut-off for low vitamin levels in the present study. However, literature and expert opinion in the field suggest that these cut-off levels may be too low, and that suboptimal vitamin levels may exist in the low-normal spectrum. Selhub *et al.*^39^ showed that homocysteine levels begin to rise at a cut-off level for vitamin B_12_ of 300 pmol/l, and at 10 nmol/l for folate. Based on these data, results below these levels were considered low normal. A combination of low levels of P-B_12_ or P-folate together with P-homocysteine (>15 μmol/l) or S-MMA (>0.34 μmol/l) levels above laboratory reference intervals was considered a deficiency.

Plasma HIV-RNA was determined using the Roche COBAS TaqMan assay version 2 (Hoffman La-Roche, Basel, Switzerland). All other blood tests were analysed according to local laboratory standards.

### Statistical analysis

Power analysis was used to determine sample size, based on difference in log plasma NfL. A sample size of 25 in each group would yield a power of 80% to detect a difference with an effect size of 0.7 (Cohen’s d) for Log NfL values at 12 months. To account for dropouts, we set 30 as the target sample size. Variables were log-transformed when suitable. Pearson correlation coefficient was used to calculate relationships. Independent sample *t*-test was used to compare differences between groups. Paired sample *t*-test was used to compare differences within groups. The tests performed were two-tailed, and *P* < 0.05 was considered significant. SPSS Statistics version 27 (IBM SPSS Statistics, Armonk, NY, USA) or Prism version 9 (GraphPad Software, La Jolla, CA, USA) were used to perform the analyses.

### Ethics approval

The study was performed in accordance with the Helsinki Declaration. All participants gave their written consent to participate in the study. The study was approved by the Research Ethics Committee at Gothenburg University (Dnr: 029-16) and the national Swedish Medical Products Agency. The study is registered at clinicaltrials.gov, NCT number: NCT02773147, and in the European Union Drug Regulating Authorities Clinical Trials Database, EudraCT number: 2015-004311-20.

## Results

From April 2016 to June 2017, 124 PLHIV were screened for participation in the study. Sixty-one of them had plasma homocysteine ≥12 μmol/l and were included in the treatment study, with 31 in the active treatment arm and 30 in the control arm (Table 1 for baseline characteristics). Three participants in the active treatment arm and three participants in the control arm discontinued the study prior to 12 months of follow up. An additional four from the active treatment arm and three from the crossed over arm discontinued before 24 months of follow up (see Supplementary Fig. 1). One patient was excluded from the study due to neurological or psychiatric disease.

**Table 1:** Baseline characteristics

	Active treatment arm	Control arm
Number (*n*)	31	30
Female (*n*)	5	8
Male (*n*)	26	22
Age median (IQR)	53 (46–62)	49.5 (45–53.75)
CD4 cell count (×10e6) median (IQR)	620 (480–820)	615 (482.5–865)
HIV-RNA (copies/ml) median (IQR)	20 (20–20)	20 (20–20)
P-NfL (pg/ml) geometric mean (GSD)	13.1 (1.70)	11.1 (1.57)
P-NfL above laboratory reference value, *n* (%)	13 (41.9)	11 (36.7)
P-homocysteine (μmol/l) mean (SD)	15.9 (±3.1)	15.4 (±2.7)
P-B_12_(pmol/l) mean (SD)	315 (±113)	312 (±147)
P-folate (nmol/l) mean (SD)	10.9 (±3.5)	12.8 (±6.2)
eGFR median (IQR)	75 (70.2–88.5)	84.85 (72.075–90.55)
Hb (g/l) median (IQR)	152 (145–160)	151 (136.5–156)
MCV (fl) median (IQR)	95 (93–100.5)	96 (91–98.5)
MADRS median (IQR)	4 (1–8)	4 (0–6.5)
AUDIT median (IQR)	3.5 (1.75–6)	2.5 (1–4)
Cogstate COMB score, mean (SD)	−0.195 (±0.469)	−0.287 (±0.546)

### Screening

**Figure 2:**
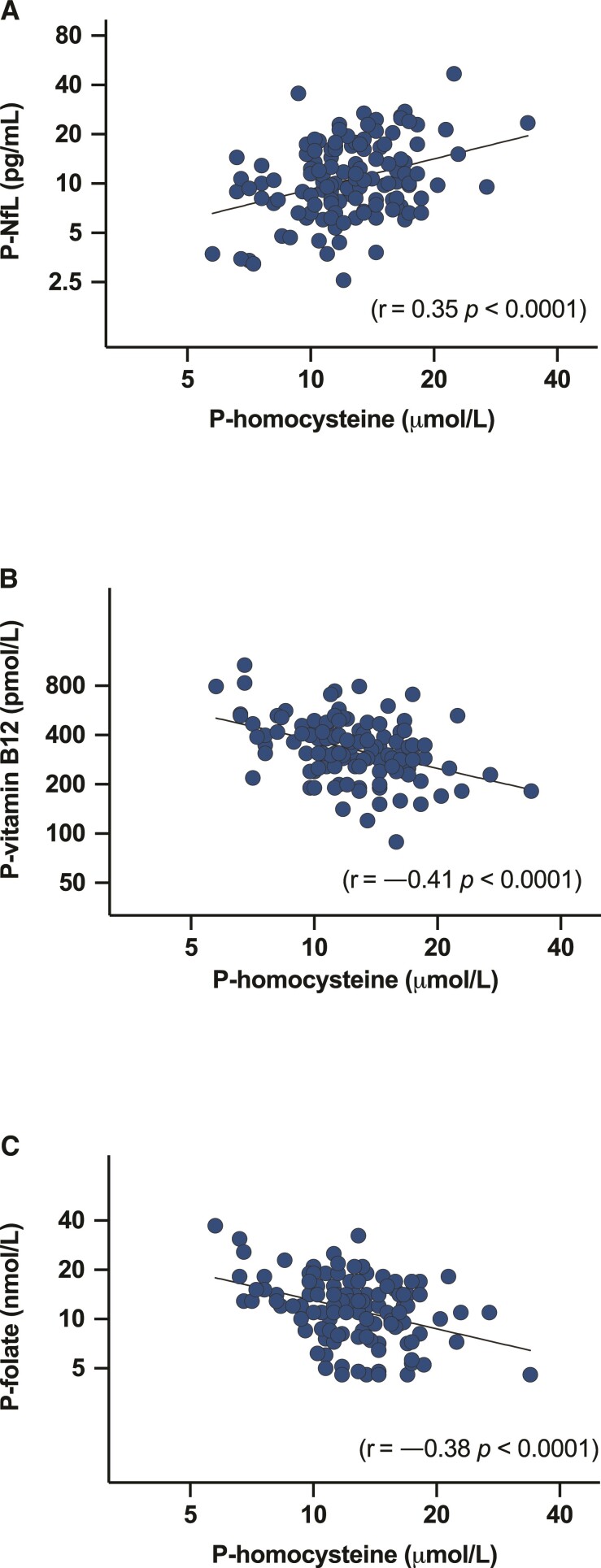
**Correlations at screening.** (**A**) Correlation between Log P-NfL and Log P-homocysteine in 124 virally suppressed PLHIV. (**B**) Correlation between Log P-homocysteine and Log P-vitamin B_12_ in 124 virally suppressed PLHIV. (**C**) Correlation between Log P-homocysteine and Log P-folate in 124 virally suppressed PLHIV.

In the screening cohort (*n* = 124), there was a significant correlation between Log P-NfL and Log P-homocysteine (*r* = 0.35, *P* < 0.0001; Fig. 2A). P-homocysteine and P-vitamin B_12_ (*r* = −0.41, *P* < 0.0001), and P-homocysteine and P-folate (*r* = −0.38, *P* < 0.0001) showed, as expected, an inverse correlation (Fig. 2B and C).

The mean P-homocysteine level was 13.0 μmol/l (SD ± 4.16). A total of 34 individuals (27.4%) had homocysteine levels above 15.0 μmol/l. The geometric mean level of P-NfL was 10.6 (GSD 1.70) pg/l. Fifty-two (41.9%) had P-NfL levels above the age-dependent laboratory norms.

### Randomized controlled trial

P-homocysteine concentrations above the UNL (>15 μmol/l) were found in 32/61 (52.5%) of the treatment study participants at baseline. Two individuals had low plasma vitamin B_12_ levels (<140 pmol/l) and another 30 had low-normal levels (<300 pmol/l). One individual was B_12_ deficient. P-folate levels were low (<7 nmol/l) in 7 subjects and low normal (7–10 nmol/l) in another 17. Four individuals were folate deficient. The geometric mean P-NfL level was 12.1 (GSD 1.64); 24 (39.3%) had P-NfL levels above age-dependent laboratory reference values (see Table 1 for distribution between groups).

#### Impact of B-vitamin supplementation on P-NfL

**Figure 3:**
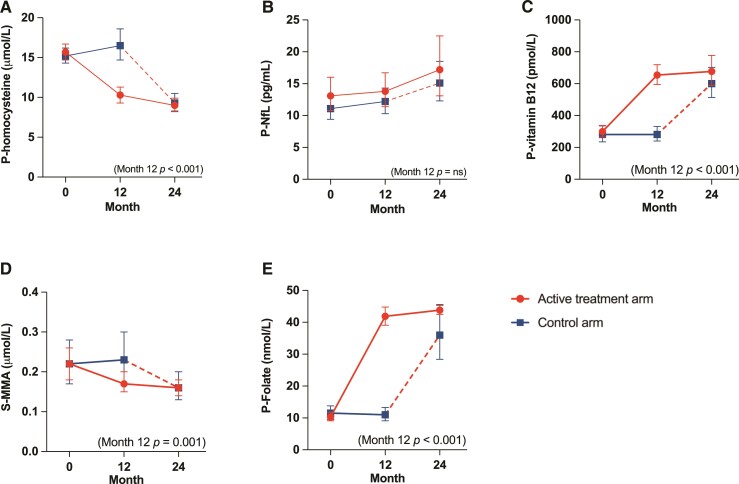
**Effect of vitamin B supplementation.** Effect of supplementation with cyanocobalamin 0.5 mg, folic acid 0.8 mg and pyridoxine 3.0 mg q.d. on (**A**) P-homocysteine, (**B**) P-NfL, (**C**) P-vitamin B_12_, (**D**) S-MMA and (**E**) P-folate in the active treatment arm for 24 months. Control arm receives no treatment during Months 0–12, and thereafter cross over to active treatment (in the figure shown as a dotted line) for Months 12–24. Variables shown as geometric mean with 95% CI. An independent *T*-test was used to calculate the effect of B-vitamin supplementation at Month 12. (**A**) *t* = −6.83, *P* < 0.001. (**B**) *t* = −0.87, *P* = 0.39. (**C**) *t* = 11.9, *P* < 0.001. (**D**) *t* = −3.66, *P* = 0.001. (**E**) *t* = 14.3, *P* < 0.001.

P-homocysteine levels decreased by 35% [95% confidence interval (CI) 28–42%] between baseline and Month 12 in the active treatment arm. This was significantly different [−40% (−48 to −30%), *P* < 0.001] from the control arm, where the P-homocysteine levels increased by 7% (−3 to 19%). P-homocysteine decreased by an additional 9% in the active treatment arm during Months 12 to 24. The P-homocysteine levels of the control group decreased by 44% from Months 12 to 24 after initiation of vitamin B supplementation (see Fig. 3A and Table 2).

**Table 2:** Effect of vitamin B supplementation

		Baseline	Month 12	Month 24
**P-homocysteine (μmol/l)**				
	Active treatment arm	15.7 (14.7–16.7)	10.3 (9.3–11.3)	9.0 (8.2–9.9)
	Control arm	15.2 (14.3–16.2)	16.5 (14.7–18.6)	9.3 (8.3–10.5)
**P-NfL (pg/l)**				
	Active treatment arm	13.1 (10.8–16.0)	13.8 (11.4–16.7)	17.2 (13.1–22.5)
	Control arm	11.1 (9.4–13.2)	12.2 (10.3–14.3)	15.1 (12.3–18.5)
**P-vitamin B_12_ (pmol/l)**				
	Active treatment arm	299 (265–338)	654 (595–719)	677 (590–777)
	Control arm	281 (235–335)	281 (240–331)	600 (513–702)
**S-MMA (μmol/l)**				
	Active treatment arm	0.22 (0.18–0.26)	0.17 (0.15–0.20)	0.16 (0.14–0.18)
	Control arm	0.22 (0.17–0.28)	0.23 (0.17–0.30)	0.16 (0.13–0.20)
**P-folate (nmol/l)**				
	Active treatment arm	10.3 (9.1–11.8)	41.9 (39.1–44.8)	43.8 (42.5–45.3)
	Control arm	11.5 (9.5–13.8)	11.0 (9.1–13.3)	36.0 (28.4–45.6)

We found no significant differences in change of P-NfL levels between the groups after 12 months of vitamin B supplementation [−6.5% (−20 to 9%), *P* = 0.39]. *P*-NfL levels increased by 1% (−10 to 14) between baseline and Month 12 in the active treatment arm, and by 8% (−1 to 19) in the control arm. From 12 to 24 months the active treatment arm increased the levels by 18% and crossed control arm by 23% (Fig. 3B and Table 2).

P-B_12_ levels increased 2.3-fold (2.1–2.6) in the active treatment arm from baseline to Month 12, and continued to increase throughout the study period. A significant difference in P-B_12_ was found between groups at Month 12 [119% (92–149%), *P* < 0.001]. In accordance, S-MMA levels decreased during the course of the study, by 23% (13–31) from baseline to Month 12. A significant difference in S-MMA was found between groups at Month 12 [−24.5% (−35 to −12%), *P* = 0.001; Fig. 3C and D and Table 2].

*P*-folate levels increased 4-fold (3.4–4.6) in the active treatment arm during the first 12 months of the study. A significant difference between groups was found at Month 12 [312% (237–403%), *P* < 0.001; Fig. 3E and Table 2].

#### Impact of B-vitamin supplementation on cognitive function

There was no significant correlation between either P-homocysteine (*r* = −0.01, *P* = 0.97) or log P-NfL (*r* = 0.06, *P* = 0.66) and the Cogstate combined *z*-score at baseline.

In addition, no significant difference was found in the Cogstate combined z-score between the groups [−0.08 (−0.33 to 0.17), *P* = 0.53] at baseline compared with Month 12 [study group: mean −0.1951 (SD ± 0.4686) versus −0.1720 (SD ± 0.5482) control group: mean −0.2871 (SD ± 0.5455) versus −0.2526 (SD ± 0.6398)]. The difference in neurocognitive performance measured by Cogstate between baseline and Month 24 in the treatment group was also not significant [0.09 (−0.06 to 0.23), *P* = 0.25]. Neither was any significance found in the individual tests at Month 12 nor Month 24. No significant differences in MADRS results were found between the groups at baseline compared with Month 12.

## Discussion

We believe our study is the first randomized controlled trial of the effect of vitamin B supplementation on markers of neuronal injury in PLHIV. As predicted, we found that treatment with vitamin B_12_, folic acid and vitamin B_6_ significantly decreased P-homocysteine levels. However, no change was found in P-NfL as a marker of ongoing neuronal injury.

In agreement with our previous results,^34^ there was a significant correlation between P-homocysteine and P-NfL concentrations. The screening cohort had a P-homocysteine mean of 13.0 μmol/l, consistent with 13.1 and 15.1 μmol/l in previous studies of PLHIV on ART.^40,41^

By way of confirmation of our findings, a study by Remacha *et al*.^42^ also showed that treatment with vitamin B_12_ and folic acid decreased the levels of P-homocysteine and increased the levels of B_12_ and folate in a cohort of PLHIV. This is a pattern well known from studies of HIV-negative elderly people with or without cognitive impairment receiving vitamin B supplementation.^43,44^

Early in the HIV pandemic, prior to the introduction of highly active ART low levels of vitamin B_12_ in serum were frequently found in PLHIV.^45,46^ One study suggested that low vitamin B_12_ may be a risk factor for disease progression to AIDS.^47^ Malabsorption due to HIV enteropathy or opportunistic intestinal infections may, at least in part, explain that finding.^48^ However, those explanations are not applicable to our cohort of PLHIV on ART with stable CD4^+^ counts and undetectable HIV-RNA, and do not explain the frequent finding of pathologically elevated homocysteine levels (>15 μmol/l) in the screening cohort (27.4%). We found vitamin B_12_ levels in the lower spectrum (<300 pmol/l) in 49% of PLHIV at baseline in the treatment study; however, only one participant had low levels in combination with elevated S-MMA/P-homocysteine, suggesting that suboptimal vitamin B_12_ levels may still be prevalent in the ART era. Nevertheless, our finding that NfL does not decrease during vitamin B_12_ supplementation, although homocysteine/MMA levels decrease and P-B_12_ increases, speaks against suboptimal vitamin B_12_ levels as the casual factor of the relationship between P-homocysteine and NfL.

Levels of P-folate in the lower spectrum (<10 nmol/l) were found in 24 (41.4%) of the study participants, which is comparable with the frequency of low-normal levels of vitamin B_12_ recorded in the same cohort. The limitation of P-folate testing is that it mirrors recent folate. Blood folate is a more accurate test of the folate depot, but is nowadays less used due to methodological difficulties.^49^ However, even though all but two participants in the treatment group normalized their P-homocysteine after 12 months of Triobe supplementation (one of whom discontinued the study after 12 months due to poor adherence), and all became folate replete; we did not see a significant decrease in NfL, thus not supporting suboptimal folate levels as the casual factor.

Less data are currently available on plasma than on CSF levels of NFL in PLHIV on stable ART. In our cohort, 39.3% had levels above laboratory norms. In addition to the CNS, NfL is also found in the peripheral nervous system (PNS) and previous studies have found elevated P-NfL in diseases affecting the PNS.^50,51^ None of the individuals included in our study had a symptomatic confounding neurological disease affecting the CNS or the PNS, suggesting that P-NfL levels reflect ongoing neuronal injury related to HIV or homocysteine metabolism. On the other hand, finding 39.3% with NfL levels above laboratory norms is higher than expected, when compared with data on NfL in CSF among PLHIV.^9^ It is possible that undiagnosed subclinical peripheral neuropathy does after all contribute to this, and that to inevitably differentiate between peripheral and central injury both plasma and CSF samples are needed. Difference in P-NfL was chosen as the primary outcome since it is a sensitive marker of neuronal injury, it reflects ongoing injury^11^ and it would make it possible to detect a change even if not clinically apparent. It has been proposed that the failure to prove the effect of vitamin B supplementation on neurocognitive symptoms in HIV-negative trials is due to the fact that the neuronal injury that gives rise to symptoms is irreversible, and therefore not affected by administration of B vitamins. However, since NfL reflects ongoing neuronal injury, the above is unlikely to be the reason for the absence of effect in the present trial.

NfL in both plasma and CSF increases with age, although the underlying mechanism is yet to be determined.^8,15^ Thus, an increase of P-NfL during the course of the study was expected. The increase we detected from baseline to Month 12 was 1 and 8% in the active treatment arm and the control arm, respectively. The modest increase of 1%, although not statistically significant, could indicate a treatment effect that compensates aging. However, the P-NfL of the control arm increased with 23% after initiating treatment, and the treatment arm increased by 18% during the next 12 months, which speaks against an effect of B-vitamin substitution on P-NfL. This was a higher annual increase than previously found in healthy HIV-negative controls (2.2–3.2%).^15,52^ It is not well established at which rate NfL increases in virologically suppressed PLHIV, when compared with HIV-negative individuals. It is well established that homocysteine is inversely correlated to renal function.^23^ Some (but not all) recent studies on PLHIV and HIV negative have found a weak association between P-NfL and renal function.^17,53,54^ It is unlikely that renal function would have had an influence on the results in this cohort of PLHIV with normal renal function.

A major limitation to our study is its open design, without placebo in the control arm. Consequently, recruitment or effect bias could not be excluded. Another limitation is the lack of CSF-NfL, imaging and neurophysiological examination, in consequence whereof subclinical CNS or PNS disease could not be excluded. In addition, the study group was relatively small: a larger cohort might have been needed to detect a change in P-NfL. The FACIT and VITACOG trials that found effects on cognitive function in HIV-negative elderly people included 818 and 266 individuals, respectively.^29,30^ While the FACIT trial had a follow-up time of 3 years, the VITACOG had the same follow-up time as the present study (2 years), which speaks against too short a follow-up time as the reason for the lack of effect. In addition, since NfL detects ongoing injury, it is reasonable to expect an effect sooner than when cognitive testing is the primary outcome. However, it is not possible to rule out the possibility that a longer course of treatment might have been beneficial. In a clinical setting, symptomatic B_12_ deficiency is treated with high oral (≥1000 μg) or intramuscular doses and folate deficiency with 5 mg q.d.^55,56^ The doses administered in our trial may have been insufficient to bring about an effect on the CNS. However, the folic acid and vitamin B_12_ dosage used have been shown to have good lowering effects on homocysteine^57,58^ and were comparable with the above-mentioned trials. It is notable that these trials consisted of cohorts of elderly subjects both with and without cognitive impairment and therefore may not be directly comparable with our cohort.

Data are scarce on the relationship between homocysteine, B vitamins on the one hand and cognitive function and neural injury in HIV infection on the other. To the best of our knowledge, only one other study has examined this relationship in virally suppressed PLHIV. Falasca *et al*.^40^ found a relationship between elevated homocysteine levels and neurocognitive performance. However, they considered ≥12 μmol/l as elevated, and since our cohort all had homocysteine levels ≥12 μmol/l, it is not possible to compare our results. We did not find an association between P-NfL or homocysteine levels and cognitive test results at baseline. However, the result of a cognitive test is multifactorial and cannot distinguish between the results of an ongoing injury and a previous one. In addition, we found that vitamin B supplementation had no effect on cognitive function. While there was little room for change in cognitive results in this group of PLHIV having no substantial neurocognitive disease, we chose to investigate them because in our previous study, we found a correlation between CSF-NfL and P-homocysteine in a group of neuroasymptomatic PLHIV.

In addition, the lack of effect may be attributable to the reasons stated above.

The present study, in addition to our previous research, shows a highly significant correlation between P-homocysteine and NfL. However, the results indicate a non-vitamin B–dependent cause. The association between NfL and homocysteine is of uncertain clinical relevance, although may constitute a piece of the puzzle in clarifying the pathogenesis of neuronal injury in virally suppressed HIV.

In corroboration of previous findings, a highly significant statistical correlation was detected between NfL and P-homocysteine. While homocysteine significantly decreased during treatment with cyanocobalamin (0.5 mg), folic acid (0.8 mg) and pyridoxine (3.0 mg) q.d., there were no changes recorded in P-NfL levels. Our results indicate a non-vitamin B–dependent association between NfL and homocysteine in HIV-related neuronal injury. Further investigation may be required to reveal the cause of this association.

## Supplementary Material

fcac259_Supplementary_Data

## Data Availability

Data is available upon request from the corresponding author.

## Abbreviations

ANI =: asymptomatic neurocognitive impairment
ART =: antiretroviral therapy
AUDIT =: alcohol use disorders identification test
HAD =: HIV-associated dementia
MADRS =: montgomery Åsberg depression rating scale
NfL =: neurofilament light protein
PLHIV =: people living with HIV
PNS =: peripheral nervous system
*P* =: plasma
S-MMA =: *S*-methylmalonic acid
UNL =: upper normal reference level.
