# Supplementary figures and images for: The effect of vitamin B supplementation on neuronal injury in people living with HIV: a randomized controlled trial
Source: Brain Commun. 2022 Oct 15;4(6):fcac259. doi: 10.1093/braincomms/fcac259 (PMC9631976; doi:10.1093/braincomms/fcac259)

## Supplementary Figure 1.

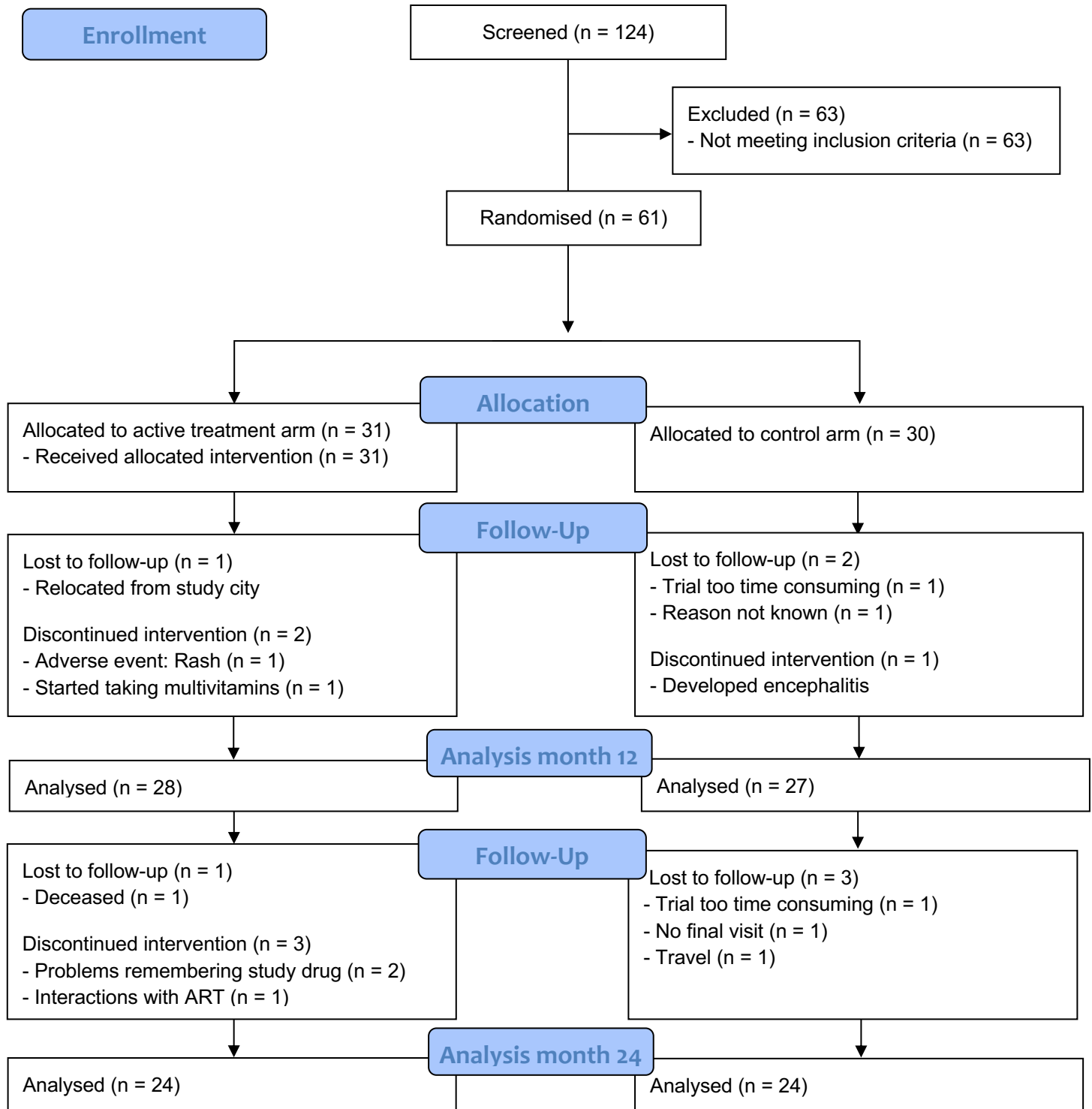

Flow chart of the study

Supplement: fcac259_Supplementary_Data [file fcac259_supplementary_data.pdf]
